# Cases Extracted from the Note-Book of Henry Davies, M.D.

**Published:** 1834-10-01

**Authors:** 


### ORIGINAL COMMUNICATIONS.

---

*Cases extracted from the Note-book of* Henry Davies, M.D., Physician to the British Lying-in Hospital, &c.

Case I. *Marasmus, with Lymphatic Tumours.*

May 1st, 1820. J. Giles, who is now twelve months old, was born a fine healthy child, was vaccinated at the age of six weeks, took the breast well, and was a robust infant at the seventh month. At this period, small tumours appeared in different parts of the body, resembling carbuncles: they made slow progress, and were not painful. Being attacked with measles, however, the eruption disappeared suddenly on the third day; the limbs then began to swell, and the tumours slowly suppurated; first a whitish, and then a greenish matter oozed from them, without any apparent diminution of bulk.

The child is now thin, the skin pale and flabby, the eyes dim, and he moans constantly; there is no appearance of teeth, or increased secretion of saliva; the pulse is somewhat accelerated. The thigh, from a tumour near the acetabulum, appears as if dislocated. The mother has taken him to several medical practitioners, and his bowels have been well cleaned out. His stools are regular, and of the natural colour. The child will take nothing but the breast.

He was ordered an antispasmodic mixture, a purge of calomel and rhubarb, and the tepid bath every night.

May 3d. Hydr. cum Cretâ gr. iij. omni nocte. Continuentur balneum, et Mist. Antispasmodica.

May 10th. The child wastes more, and is extremely emaciated; his eyes are brighter; there is no appearance of teeth; the temperature of the skin is low. He perspires copiously; the urine is turbid, and stains his linen. He was ordered a nourishing diet, with wine and beef-tea, and to have the following ointment rubbed in: ℞. Camphoræ ʒi.; Antim. Tart. gr. v.; Ung. Hydrarg. mit. ʒij. M. ut fiat unguent. cujus quarta pars dorso quotidie infricetur.

14th. The general symptoms are as in the last report. A tumour is forming on his finger, and the one on the thigh is discharging a thin matter. He vomits after taking the breast.

24th. Looks livelier; stools more frequent; sleeps ill.—℞. Hydr. Subm., Pulv. Antim. āā gr. ss. M. ut fiat pulv.

h. s. s.—℞. Opii pulv. ℈i.; Antim. Tart. gr. x.; Axungiæ ℥ss. M. ut fiat ung. cujus quarta pars dorso infricetur o. n. Continuetur balneum.

28th. The bowels are quieter. He does not throw up the breast-milk, but, if he is made to take anything else, vomiting and violent spasms are the consequences. He sleeps better since the opiate friction.

June 2d. He rests better; there is a pustular eruption on his back; the tumours are diminished in size.—Continuentur medicamenta.

June 8th. Continues to waste; takes the breast well, but not anything else.

June 20th. Died exhausted.

*Dissection.* The intestines were empty, and the arch of the colon was filled with flatus, distending the abdomen; the mesenteric vessels were somewhat turgid, and the glands slightly enlarged. The stomach was much contracted, but not diseased in its structure. The lungs were tuberculated throughout.

The tumours were encysted, and contained a curdy matter.

Dr. Cheyne mentions the occurrence of tumours in cases of hydrocephalus. They were of the size of a nutmeg, and apparently situated in the cellular texture, suppurated without pain, and left behind scrofulous sores. He says that he has never known these tumours except in constitutions where there was a strong tendency to scrofula, and that they are not dangerous unless numerous. Recovery, however, seldom takes place: some new form of scrofula appears, and the patients die tabid.

I have, however, seen two cases, in which the recovery was perfect. Miss C., when two years old, suffered from a protracted cough, marasmus, and several of these tumours: she is now a fine healthy girl of fifteen; her mother died consumptive. Miss G. had these tumours, of which one was large, and at the insertion of the deltoid. The remains of the tumour are still to be seen; but this patient likewise is now healthy.

In the case of Giles, as he was of an age when he ought to have had teeth, I endeavoured to procure artificial salivation, to produce counter-irritation, and to give him temporary support by stimulants. In consultation, generous living was advised; but his stomach resisted it. It is probable that the recession of the measles had affected the lungs.

Case ii. (Reported by Mr. Laidlaw.) *Scrofula.*

July, 1826. The subject of the following dissection was about five years of age, of a fair complexion, and delicate habit. The disease had existed between two and three years. The symptoms were, latterly, emaciation, debility, slight cough, and difficulty of breathing, but no expectoration.

*Sectio Cadaveris, sixty-five hours after death.* Skin of a mottled green colour; emaciation considerable, but greater on the thorax than the abdomen.

*Thorax.* The lungs on both sides adhered to the pleura costalis, and also to the diaphragm; they were of a mottled, dark, blackish colour, when cut into; tubercles were found to pervade their whole substance; several of them were two-thirds of an inch in diameter, and one of them, at the apex of the left lobe, communicated by an aperture with the cavity of the thorax. At the superior part of the right lobe, where the bronchi enter, two concretions were found. There were about five ounces of fluid.

*Abdomen.* On exposing the viscera, the intestines, which were red and much inflated, were found to have formed extensive adhesions with the peritoneum. They were so vascular, that, in several parts, the whole circumference of the intestine assumed the appearance of a well-injected preparation.

*Mesentery.* Its glands were enlarged throughout, several exceeding a pigeon's egg in size. When cut into no one was found to have suppurated, or become scirrhous, but they were of a doughy consistence.

*Spleen.* The adhesions of this organ with the stomach were so strong as to be separated with great difficulty. These adhesions were probably formed by the irritation communicated from the mesentery and the intestines in its vicinity, which were more inflamed here than in any other part; for, when the spleen itself was cut into, its substance was found to be quite natural.

*Stomach.* The stomach contained a considerable quantity of fluid, but, so far as it was examined, was in a healthy state.

*Liver.* The liver had formed extensive adhesions with the diaphragm, which appear to have been produced by the irritation communicated from the lungs. The disease was merely superficial; for, when cut into, the substance of the liver was healthy.

*Gall-bladder.* This viscus was two-thirds full of yellow bile, and was in every respect perfectly healthy.

The cavity of the abdomen contained about half a pint of bloody serum.

